# Application of Artificial Intelligence in COVID-19 Diagnosis and Therapeutics

**DOI:** 10.3390/jpm11090886

**Published:** 2021-09-04

**Authors:** Ken Asada, Masaaki Komatsu, Ryo Shimoyama, Ken Takasawa, Norio Shinkai, Akira Sakai, Amina Bolatkan, Masayoshi Yamada, Satoshi Takahashi, Hidenori Machino, Kazuma Kobayashi, Syuzo Kaneko, Ryuji Hamamoto

**Affiliations:** 1 Cancer Translational Research Team, RIKEN Center for Advanced Intelligence Project, 1-4-1 Nihonbashi, Chuo-ku, Tokyo 103-0027, Japan; ken.asada@riken.jp (K.A.); maskomat@ncc.go.jp (M.K.); r.shimoyama.oums@gmail.com (R.S.); ktakazaw@ncc.go.jp (K.T.); norio.shinkai@riken.jp (N.S.); abolatka@ncc.go.jp (A.B.); satoshi.takahashi.fy@riken.jp (S.T.); hmachino@ncc.go.jp (H.M.); kazumkob@ncc.go.jp (K.K.); sykaneko@ncc.go.jp (S.K.); 2 Division of Medical AI Research and Development, National Cancer Center Research Institute, 5-1-1 Tsukiji, Chuo-ku, Tokyo 104-0045, Japan; dr200004@tmd.ac.jp (A.S.); masyamad@ncc.go.jp (M.Y.); 3 Department of NCC Cancer Science, Graduate School of Medical and Dental Sciences, Tokyo Medical and Dental University, 1-5-45 Yushima, Bunkyo-ku, Tokyo 113-8510, Japan; 4 Department of Endoscopy, National Cancer Center Hospital, 5-1-1 Tsukiji, Chuo-ku, Tokyo 104-0045, Japan

**Keywords:** COVID-19, artificial intelligence, diagnosis, therapeutics, public health

## Abstract

The coronavirus disease 2019 (COVID-19) pandemic began at the end of December 2019, giving rise to a high rate of infections and causing COVID-19-associated deaths worldwide. It was first reported in Wuhan, China, and since then, not only global leaders, organizations, and pharmaceutical/biotech companies, but also researchers, have directed their efforts toward overcoming this threat. The use of artificial intelligence (AI) has recently surged internationally and has been applied to diverse aspects of many problems. The benefits of using AI are now widely accepted, and many studies have shown great success in medical research on tasks, such as the classification, detection, and prediction of disease, or even patient outcome. In fact, AI technology has been actively employed in various ways in COVID-19 research, and several clinical applications of AI-equipped medical devices for the diagnosis of COVID-19 have already been reported. Hence, in this review, we summarize the latest studies that focus on medical imaging analysis, drug discovery, and therapeutics such as vaccine development and public health decision-making using AI. This survey clarifies the advantages of using AI in the fight against COVID-19 and provides future directions for tackling the COVID-19 pandemic using AI techniques.

## 1. Introduction

The coronavirus disease 2019 (COVID-19), which was confirmed in Wuhan, Hubei Province, People’s Republic of China, in December 2019, declared a Public Health Emergency of International Concern on 30 January 2020, and declared a pandemic on 11 March 2020, by the World Health Organization (WHO), poses a public health threat to people worldwide [1,2,3]. Several vaccines against COVID-19 have been rapidly developed, and vaccination, which is expected to calm the situation, is already underway around the world [4,5]. However, many variants of SARS-CoV-2 that cause COVID-19 have been reported, and this situation remains unpredictable [6]. It has been pointed out that among these SARS-CoV-2 variants, there may be variants that increase infectivity and transmissibility, alter antigenicity, and even reduce immunity and vaccine efficacy [7,8,9,10]. The development of therapeutic agents for COVID-19 is also being actively pursued, and in fact, drugs such as remdesivir, dexamethasone, and baricitinib have been approved and are being used in clinical practice [11,12]. However, current treatment for COVID-19 is mainly symptomatic, and although several experimental therapies are being actively studied in clinical trials, in some cases, there is insufficient high-quality evidence to recommend early treatment [13,14]. In particular, hydroxychloroquine and lopinavir/ritonavir, which were thought to be promising, have been found to be ineffective and even harmful in subsequent studies [13,14]; hence, it is necessary to be cautious and patient when considering the therapeutic effects of drugs. In view of the above, stringent responses and countermeasures against COVID-19 will continue to be an important public health issue worldwide.

In recent years, with the rapid progress of machine learning (ML) techniques, especially deep learning (DL), the emergence of inexpensive and high-performance graphics processing units (GPUs), and the expansion of public databases, the expectations for artificial intelligence (AI) are increasing worldwide [15]. The medical field is no exception, and many AI-powered medical devices have been developed and are now being applied clinically [15]. In medical research, AI has been introduced into various tasks, including medical image analysis, such as radiological image analysis, endoscopic image analysis, and pathological image association analysis; omics analysis such as genome analysis, epigenome analysis, and proteome analysis; and natural language processing for drug discovery, electronic medical record information, and literature search [16,17,18,19,20,21,22,23,24,25,26,27,28,29,30,31,32,33]. Importantly, research on COVID-19 is being conducted worldwide, and AI is now being actively used in vaccine development, the development of new diagnostic methods, and the development of new therapeutic agents by extracting important features from vast amounts of data. In this review, we review the current status of AI applications in the development of vaccines against COVID-19, new diagnostic methods, and new therapeutic agents, and discuss the importance of AI in dealing with COVID-19.

## 2. Medical Imaging Analysis

**Figure 1:**
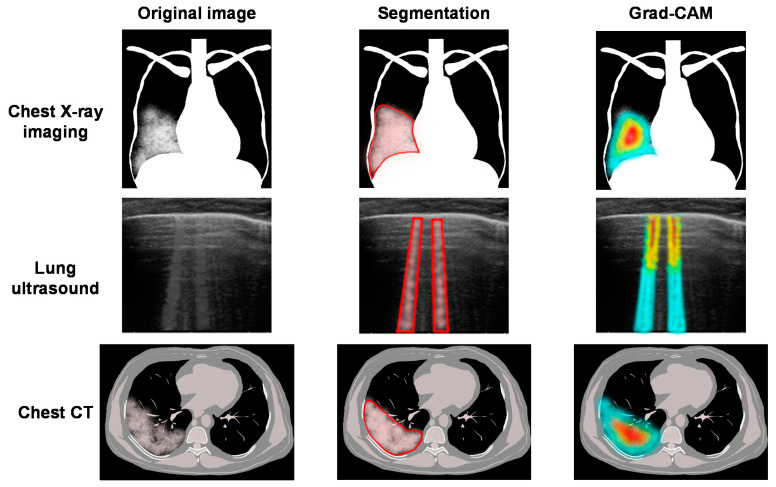
Segmentation and the gradient-weighted class activation mapping (Grad-CAM) have been used in various AI-based medical imaging analyses in COVID-19. Grad-CAM provides a clinically interpretable saliency map that indicates the discriminative regions of the image that determine the classification of the severity of COVID-19.

In this section, we focus on chest and lung imaging analyses using AI. The WHO has developed a rapid guide on the use of chest imaging for the diagnosis and management of COVID-19 [34]. The relevant chest imaging modalities include chest radiography (X-ray imaging), chest computed tomography (CT), and lung ultrasound. Chest X-ray imaging and lung ultrasound can be performed using portable equipment at the point of care. Chest CT has the highest sensitivity, but relatively low specificity, and can be useful in patients with pre-existing pulmonary diseases. The differential diagnoses and potential complications for each specific case (e.g., CT angiography for pulmonary thromboembolism and cardiac ultrasound for myocarditis) should be considered when choosing the imaging modality. During the global COVID-19 pandemic, numerous efforts have been made regarding AI-based medical imaging analysis of COVID-19 (Figure 1).

A DL framework for COVID-19 detection on chest X-ray images was reported that fine-tunes four pre-trained convolutional models (ResNet18, ResNet50, SqueezeNet, and DenseNet121) on a limited training dataset [35]. To address the difficulty of systematic data collection of chest X-ray images in patients with COVID-19, a patch-based convolutional neural network (CNN) approach with a relatively small number of trainable parameters for COVID-19 diagnosis on chest X-ray images was proposed. This method uses gradient-weighted class activation mapping (Grad-CAM) and provides a clinically interpretable saliency map tailored to the local patch-based approach [36]. A weakly supervised learning architecture for predicting a multi-regional score on chest X-ray images was designed to effectively and reliably assess the severity of lung compromise in patients with COVID-19 [37]. In terms of clinical management, it has been reported that the DenseNet121 model can accurately predict the need for mechanical ventilation early in the hospitalization of patients with COVID-19 using chest X-ray images [38]. A DL-based model for classifying the severity of and monitoring COVID-19 was proposed. The outputs of the different layers of the CNN under dominant filters provide valuable insights into the subtle patterns in chest X-ray images. This approach can be used to study disease progression and its influencing factors in a single patient [39].

Ultrasound is an option for real-time point-of-care testing without radiation exposure in various medical fields [30]. Although lung ultrasound requires closer physical proximity of the examiner to the patient, the risk of COVID-19 cross-infection can be minimized when a plastic disposable cover and individually packaged ultrasound gel on a portable probe are used [34,40]. B-lines are vertical comet-tail-shaped artifacts that reflect various pathological conditions of the lung [41]. B-lines and irregular pleural lines in lung ultrasound are not specific but are related to COVID-19 pneumonia. Despite the clinical significance of lung ultrasound, only a few studies have focused on AI-based lung ultrasound image analysis for COVID-19. The DL-based method, derived from spatial transformer networks, was shown to simultaneously predict the disease severity score associated with an input frame and localize pathological artifacts [42]. An integrated autoencoder-based hybrid classification model combining CNN and long short-term memory (LSTM) showed promising improvements in the prediction of COVID-19 severity using lung ultrasound frames [43]. A contrastive learning approach for assessing COVID-19 severity from lung ultrasound and clinical information was proposed that matches the two spaces while retaining the discriminative features. This method provides an interpretation of the severity assessment by grading each lung zone and identifying the pathological patterns [44]. In addition, myocarditis and cardiomyopathy can occur during COVID-19 [45]. Therefore, AI-based cardiac ultrasound image analysis is expected to improve the diagnostic accuracy of these cardiac complications at the point of care [30].

Several AI-based imaging analyses using cohorts of thousands of patients have been reported. Using a large database of chest CT scans from 3777 patients, an AI system that can diagnose COVID-19 pneumonia and differentiate it from other common forms of pneumonia and normal controls was developed. A good correlation between the COVID-19 lung lesions as determined by CT parameters and the clinical and biochemical markers of multiple organs has been observed [46]. Harmon et al. showed that a series of DL algorithms delivered acceptable performance metrics for the classification of chest CT images for COVID-19 infection using a diverse multinational cohort of patients with and without COVID-19 [47]. A model using U-Net and ResNet152 achieved accurate performance for a challenging multi-class diagnosis task from COVID-19, influenza A/B, non-viral community-acquired pneumonia, and non-pneumonia subjects [48]. Using holistic patient information, including chest CT, vital signs, blood tests, and demographic data, an ML-based model can help assess the disease burden and forecast meaningful patient outcomes with a high predictive accuracy in patients with COVID-19 pneumonia [49]. A weakly supervised deep active learning framework called COVID-AL was proposed for COVID-19 diagnosis [50]. A deep attention-based multiple instance learning framework for COVID-19 severity assessment using chest CT images via data augmentation and self-supervised learning was developed [51]. Compared with 11 existing severity scores, the multimodal AI-severity score, which includes five clinical and biological variables in addition to the DL model trained by the chest CT scan data, significantly improved the performance of prognosis for patients with COVID-19 [52]. Deep multitask learning improves the joint task of COVID-19 identification and quantification of its severity [53]. Using CT imaging and clinical data, a DL model successfully predicted the time until progression to critical illness in individual patients while identifying high-risk patients [54]. Deep supervised learning with a self-adaptive auxiliary loss (DSN-SAAL) was demonstrated to be effective for the diagnosis of COVID-19 with varying degrees of data imbalance [55]. The DL-based radiomics features of pulmonary opacities on chest CT images were superior to subjective assessments in differentiating patients with favorable and adverse outcomes [56].

## 3. Drug Discovery and Vaccine Development

### 3.1. General Background for the Vaccine

In general, there are two main approaches to fighting the worldwide COVID-19 pandemic: a vaccine-based pipeline for prevention and a chemical-based pipeline to cure infected patients. For the vaccine-based approach, at the time of writing this review article, the total number of candidate vaccines has reached 292, 108 of which are now undergoing clinical trials. Currently, 38 are in phase 1, 28 in phase 1/2, 10 in phase 2, 9 in phase 2/3, 19 in phase 3, and 8 in phase 4 as summarized by the WHO [57]. The nonprofit organization Our World in Data, established by the University of Oxford teams, stated that more than 28.5% of the world population had received at least one COVID-19 vaccine as of 3 August 2021 [58]. Under the current circumstances, the majority of the vaccinated public has been vaccinated by the two mRNA-based vaccines, BNT162b2 (Pfizer, New York, NY, USA) and mRNA-1273 (Moderna, Cambridge, MA, USA), which have been authorized for emergency use by the U.S. Food and Drug Administration (FDA). A study conducted between 4 May 2020 and 22 June 2020, showed that the response of BNT162b2, which encodes a full-length membrane-anchored spike (S) protein of SARS-CoV-2, and the response of BNT162b1, which encodes a secreted trimerized SARS-CoV-2 receptor-binding domain, are similar with respect to factors such as S1-binding IgG concentration and 50% neutralization titer. However, BNT162b2 yields a lower incidence and severity than BNT162b1, particularly in older adults [59]. In addition, BNT162b2 can neutralize several SARS-CoV-2 variants [60]. COVID-19-convalescent individuals with or without mRNA vaccines were investigated for the follow-up cohort study, which indicated that the neutralization activity against SARS-CoV-2 one year after infection was stable in the non-vaccinated group, but enhanced in vaccinated groups in terms of the components of the immune system, such as the plasma IgG antibody that can bind to the SARS-CoV-2 receptor-binding domain (RBD), N protein, and plasma neutralizing activity [61]. It was noted that the data from the seven vaccines and convalescent cohorts show that the neutralizing activity can predict immune protection from SARS-CoV-2 infection. The model indicates that protection from severe disease can be retained, but protection from SARS-CoV-2 infection would decrease [62]. Regarding mRNA-1273, efficacy and safety have been investigated, and a 94.1% efficacy in preventing COVID-19 has demonstrated [63]. The use of mRNA-1273 and BTN162b2 in pregnant and lactating women has also been examined, indicating that women can acquire immunogenicity under these conditions [64]. Similar to BNT162b2, the neutralizing activity persists for at least six months after mRNA-1273 vaccination [65], and miRNA-1273-elicited antibodies were more targeted towards SARS-CoV-2 RBD than naturally elicited antibodies [66].

### 3.2. AI-Driven Drug Discovery

Although encouraging evidence about the ability of the abovementioned mRNA-based vaccines to prevent COVID-19 has accumulated, there is still a demand for new vaccines or drugs for the following reasons: (1) Vaccines that can be stored at room temperature or even in a freezer are needed. BNT162b2 needs to be stored at −90 to −60 °C and mRNA-1273 needs to be stored at −25 to −20 °C. It is a difficult technological challenge, especially in low-income countries, to gather a sufficient amount of the necessary equipment such as deep freezers with power supplies. Hence, the development of such vaccines would accelerate an effective and strategic COVID-19 vaccination program for global mass immunization. (2) Vaccination for COVID-19 causes certain unavoidable side effects [67]. Therefore, vaccines that lead to fewer side effects are desirable. (3) There are four variants of concern (Alpha, Beta, Gamma, and Delta), which are recognized as SARS-CoV-2 virulent variants and four variants of interest (Eta, Iota, Kappa, and Lambda), which have been detected in multiple countries or cause clusters. These variants have been categorized by the WHO [68], and thus far, limited studies have investigated whether currently available vaccines are useful against them [69,70,71]. Furthermore, some patients have become infected after vaccination [72], and reduced vaccine efficacy has been reported against some variants [73].

**Figure 2:**
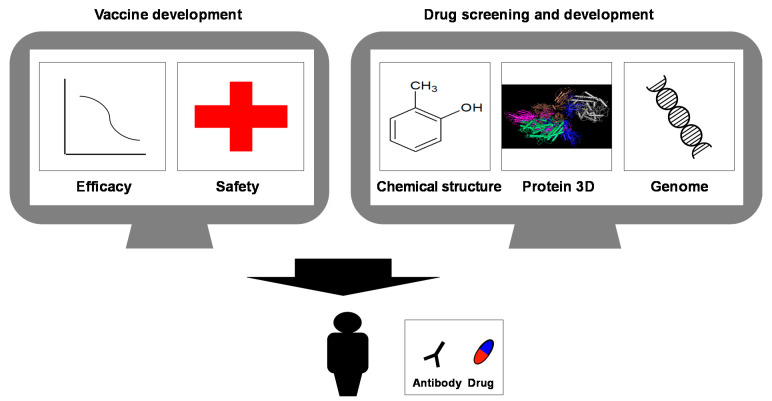
AI and COVID-19 therapeutics. Computational analysis is used for both vaccine development and chemical drug development. Prediction of vaccine efficacy, half-life, and safety can be analyzed in vaccine development. For drug development, publicly available or crowdsourced datasets are used to accelerate target screening and candidate compound design.

Exploratory studies for vaccines and drug discovery to tackle COVID-19 are actively ongoing. A DL approach can identify therapeutic candidate antibodies by predicting antigen specificity [74]. Major histocompatibility complex analysis with recurrent integrated architecture (MARIA) was trained on HLA-DR ligands identified by mass spectrometry-based profiling, public HLA-II peptide binding data (IEDB), and gene expression levels to predict potential epitopes [75]. Fast et al. further expanded this method to identify T-cell epitopes for SARS-CoV-2 RBD [76]. Antibody-epitope classification using deep neural networks (DNNs) was reported using input two-dimensional images generated from a three-dimensional image projection created by the Rosetta antibody software [77]. The ML platform REDIAL-2020 estimates small compound activities in a broad range of SARS-CoV-2-related assays [78]. ML can predict activity from chemical structures, and an ML-based drug discovery pipeline was developed to identify effective therapeutic drugs for COVID-19 from FDA-registered and approved drugs and purchasable chemicals [79]. Note that to target SARS-CoV-2 with AI, freely available datasets are deposited at the nCov-Group Data Repository [80]. In silico drug discovery with tensor decomposition-based unsupervised feature extraction was performed on lung cancer cell lines infected with SARS-CoV-2 and successfully screened for chemical compounds such as ivermectin, which is undergoing a clinical trial for SARS-CoV-2 [81]. The fragment-based drug discovery approach facilitates the identification of lead compounds, and their crystallographic screening approach identified 71 hits [82] (Figure 2).

For drug repurposing or repositioning, protein–protein interaction networks have been identified by expressing 26 out of 29 SARS-CoV-2 proteins in human cells [83] and two high-throughput repurposing screenings using HeLa cells expressing ACE2 and lung epithelial Calu-3 cell lines [84]. To screen anti-viral drugs for COVID-19, matrix completion techniques have been used to predict the drug–virus association for drug repositioning using a manually curated comprehensive dataset [85]. Another study showed that an AI platform with two different training datasets identified existing drugs with potential [86], indicating that the AI approach for drug screening is now feasible. COVID-19 mortality prediction using ML and DL in USA, China, and Korean cohorts has surged recently [87,88,89,90,91]. The COVID-19 Moonshot project, which united academic institutions such as Oxford, Imperial College London, and Memorial Sloan Kettering with industry partners, was launched to specifically focus on developing the SARS-CoV-2 main viral protease (M^pro^), which is known to be a good candidate for antivirals owing to its distinctiveness from host proteases [92]. The Moonshot project combines crowdsourcing medicinal chemistry insights with high-throughput crystallography, a computational chemistry environment, and ML for drug development to actively determine drug candidates [93,94].

## 4. Public Health

### 4.1. AI Used in Public Health Decision-Making

Public health is defined as a population-based approach to dealing with various health-related problems in human society. It serves as a framework for considering people as a group and aids in the development of measures from the town to city level and, possibly, at national level [95]. When new epidemics occur in the era of information technology, a new approach to public health that consists of “collective, coordinated, and organized activities to continuously improve the health imbalance of the local population” has been proposed [96].

COVID-19 has morphed into a global pandemic with over 182 million confirmed cases worldwide in more than 200 countries as of 2 July 2021 [97]. This may be due to people intentionally or inadvertently violating policies or infecting others without being detected as infected themselves, resulting in the rapid spread of mild cases worldwide and an increasing number of deaths [98,99]. The progress of the infection has also varied from country to country. In some cases, it has appeared to be under control, whereas in others, it reached a catastrophic scale [100]. To effectively manage this pandemic, policymakers, clinicians, and other stakeholders need access to near real-time data and recommendations, including models to assess the relative risks and benefits of various interventions. While predicting epidemics and pandemics is exceptionally complex and challenging, obtaining reliable estimates of morbidity and mortality is essential for decision-making at individual and organizational levels [101]. However, many existing prediction models may be inaccurate for this novel pathogen. In addition, given globalization, similar infections may occur in the future [102]. To address this situation, there has been much interest in developing new systems that integrate AI techniques, which have been advancing rapidly in recent years. Here, we present how AI is being used to (1) predict the dynamics of infectious diseases and the effects of interventions, (2) perform outbreak detection and surveillance, and (3) detect infectious diseases in real time, as described in reports including the latest preprints (Table 1).

**Table 1:** AI approaches used to predict the dynamics of infectious diseases and the effectiveness of interventions, to detect and monitor outbreaks, and to detect infectious diseases in real time.

Tasks of ML Models	Models Used in the Study	References
Determine a new daily cases peak with a forecasted curve	Modified autoencoder and SEIR compartment model	Distante et al. [103]
Forecast the spread of infection	First-principles epidemiological equations and neural network model	Dandekar et al. [104]
Detect early warning indicators (EWIs)	Neural network model	Uhlig et al. [105]
Long-term prediction and estimation of the number of asymptomatic infections	ML-based fine-grained simulator (ML-Sim)	Yu et al. [106]
(1) Predict new confirmed cases, (2) predict how many cases end in death, and (3) provide joint predictions of cases, deaths, and recoveries	Bayesian time series model and a random forest algorithm within an epidemiological compartmental model	Watson et al. [107]
Predict the strength and timing of the peak of the COVID-19 epidemic in Iran and the total number of cases expected during the epidemic	(1) Random forest, (2) multi-layer perceptron, and (3) LSTM	Kafieh et al. [108]
Generate forecasts of disease outbreak	PNN + cf	Fong et al. [109]
Predict the COVID-19 infection status in various regions and countries of the world	Variational LSTM autoencoder model	Ibrahim et al. [110]
Predict the number of confirmed cases in the short term	Adaptive neuro-fuzzy inference system using an enhanced flower pollination algorithm and salp swarm algorithm	Al-Qaness et al. [111]
Regression of the daily infection cases over the coming 24 days	XGBoost and MultiOutputRegressor	Suzuki et al. [112]
Combine health, demographic, and geographic characteristics to predict the near-future infection risk at county level	Three-stage XGBoost modeling process	Mehta et al. [113]
Early identification of the spread of COVID-19	DNN classifier using pre-trained bidirectional encoder representations from transformers (BERT)	Klein et al. and Golder et al. [114,115]
Identify abnormalities in the incidence of the disease	Determine the parameters that minimize mean absolute error	Chamberlain et al. [116]
Predict of influenza-like illnesses	Importance contribution index for various feature selection and pattern classification approaches	Pei et al. [117]
Ultra-fast COVID-19 virus genome signature analysis with the alignment-free approach	Supervised ML with digital signal processing	Randhawa et al. [118]
Detect fever and cyanosis; estimate heart rate and respiratory effort	Person detection using algorithms based on DL	Hegde et al. [119]
Distinguish COVID-19 coughs from non-COVID-19 coughs	Domain recognition AI engine	Imran et al. [120]
Estimate the probability that an individual will test positive for COVID-19 based on the responses to nine simple questions related to SARS-CoV-2 infection	Logistic regression models and gradient boosting decision trees models	Shoer et al. [121]

### 4.2. Models for Predicting the Dynamics of Infectious Diseases and the Effects of Interventions

**Figure 3:**
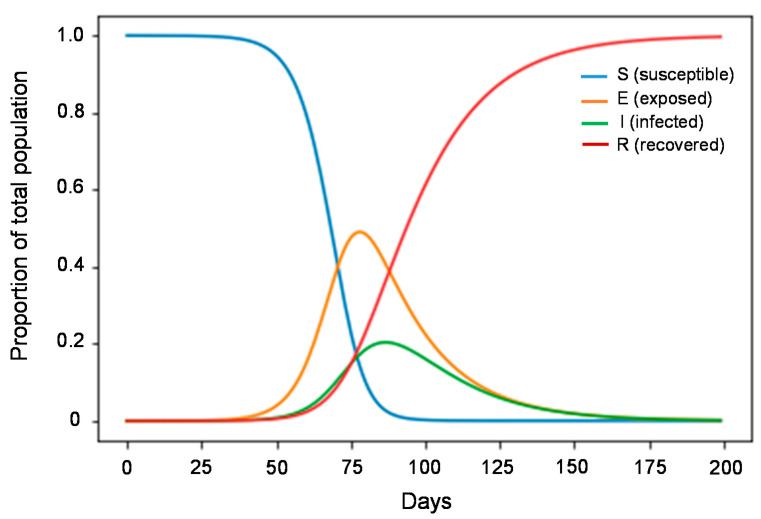
SEIR model to predict disease spread. S is the number of susceptible individuals, E is the number of exposed individuals, I is the number of infected individuals, and R is the number of recovered individuals. This model was generated using a Python script [123].

Existing models of COVID-19 can be broadly classified into mathematical models that simulate diseases in a population (such as epidemiological compartment models) and statistical curve-fitting models that estimate the future by fitting functions to observed data. Most models of COVID-19 are compartmental models that have been used by epidemiologists for more than a century to simulate infectious disease epidemics, such as the susceptible-infectious-recovered model and the susceptible-exposed-infectious-recovered (SEIR) model [122] (Figure 3).

Although compartmental models are necessary for understanding the mechanisms that occur during an epidemic, it is unknown whether they are suitable for predicting prognosis [124].

Existing models that have analyzed the role of travel restrictions in COVID-19 spread include parameters based on prior knowledge of SARS/MERS coronavirus epidemiology [125]. Several COVID-19 models have also been developed and adapted from influenza pandemic models to simulate individuals in a population and their interactions [126]. However, this can cause the model to fail because model performance depends on the assumptions and data used to “train” the model, leading to the risk of false predictions and large, unreliable uncertainty intervals. Therefore, to overcome these challenges, ideas have been devised to augment traditional models when data are limited.

COVID-19 infection prevention in various regions of China served as a valuable training model because it provided complete data, showing that the epidemic could be controlled by social distancing [127]. Distante et al. applied a modified autoencoder for time-series forecasting using data from different regions of China as training data [103]. Dandekar et al. augmented a first-principles epidemiological model with a data-driven module implemented as a neural network to analyze the control of the effective reproduction number of viruses and quarantine control policies of different countries [104]. Similarly, Uhlig et al. examined the effectiveness of measures taken by governments in different regions while updating region-specific early warning indicators (EWIs) using neural networks [105]. Yu et al. proposed a fine-grained simulator (MLSim) approach based on ML that predicts viral infections more accurately and helps estimate the number of asymptomatic patients [106]. Watson et al. also used a data analysis method that combines Bayesian and ML methods to predict the number of COVID-19 infections and deaths and learn the transition function of the compartmental model [107]. The case and death models are merged by incorporating them into a compartmentalized model that can also predict active cases and confirmed recovered cases.

By contrast, attempts to augment sparse data have also been reported. Kafieh et al. augmented data on the number of infected people in Iran over a short period of time, which only included a few weeks, with publicly available data from around the world [108]. Fong et al. argued that prediction models built with a polynomial neural network with corrective feedback (PNN + cf) are capable of low-error prediction during critical periods of disease development when samples are not abundant [109].

Large-scale data processing, real-time data sources, and various qualities are important aspects to consider when dealing with large amounts of data related to COVID-19. For example, comparisons of analysis methods based on health statistics, demographics, multiple public sources including geographic characteristics, government measures applied to each country, urban factors, and dependencies among different countries are planned [110,111,112,113]. These analyses will be useful in promoting behavioral changes among residents of at-risk areas, assisting in decisions to resume economic activity in each region, supporting decisions about infectious disease control in each region, and searching for factors that are highly correlated with future increases in the number of COVID-19 cases.

### 4.3. Surveillance and Outbreak Detection

Examples of social networking data collection and analysis include attempts to use Twitter and other social media mining methods to detect the spread of COVID-19 in the USA and UK [114,115]. Both use natural language processing and ML frameworks, which may be useful for analyzing the temporal and geographic extent and types of symptoms in different populations and regions.

If hotspots of infection can be quickly identified by collecting and analyzing health application data, the spread of infection can be controlled with limited public health resources. Chamberlain et al. used data collected from a network of personal thermometers connected to smartphones to classify and track users with elevated body temperatures over multiple days as having influenza-like illnesses [116]. The results showed that the detection of abnormal influenza-like illness outbreaks correlated with COVID-19 positive cases and was helpful in the rapid detection of COVID-related disease outliers.

With regard to the use of electronic medical record data for syndromic (influenza-like) surveillance, the emergency department information system (EDIS) can monitor the spread of influenza viruses, and electronic medical record data may be useful for the early detection of COVID-19 [117].

Genome sequencing can facilitate the classification and contact tracing of the COVID-19 viral genome. These genomic data need to be elucidated as early as possible for strategic public health planning, containment, and treatment. Recent reports have shown that ML using unique genomic signatures can be used to rapidly classify novel pathogens without alignment and may facilitate contact tracing in the future [118,128].

### 4.4. Scalable Real-Time Screening Tools

The significant increase in emergency department visits associated with the COVID-19 pandemic has placed a heavy burden on healthcare providers, and the decreased efficiency of emergency departments increases the risk of infectious diseases. Hegde et al. used a Raspberry Pi-based low-cost computer vision system to evaluate its accuracy as a triage tool [119]. Although many challenges remain, this system provides a starting point for future automated triage systems and has clear advantages in infection prevention through rapid, non-contact screening.

The idea of using cough sounds as a preliminary diagnosis of respiratory syndromes has been proposed [129,130,131,132,133,134,135,136]. Imran et al. have shown that cough can be used as a test medium for diagnosing various respiratory diseases using AI and developed a domain-aware AI engine to distinguish between coughs caused by COVID-19 and non-COVID-19 [120]. This has the potential to provide remote screening to reduce the burden on healthcare systems worldwide.

In addition, an attempt is being made to build a model to estimate the probability of being COVID-19 positive based on a simple online question, which can be used without touching a suspected infected patient, suggesting that it could be useful as a COVID-19 control measure [121,137].

## 5. Discussion

In this review, we presented recent global trends in the use of AI for the diagnosis and treatment of COVID-19. AI techniques have been actively introduced into medical image analysis, partly because of their superior performance. Moreover, AI is being used in various ways to diagnose COVID-19, mainly in chest X-ray imaging, chest CT, and lung ultrasound examinations. In fact, there are several AI-equipped medical devices that are being used for diagnostic imaging of COVID-19 patients in clinical practice, and it is expected that additional research results will continue to be applied clinically. AI has also been introduced into COVID-19 vaccine development and drug discovery; examples include the prediction of antigen specificity using DL and the prediction of the activity of small molecule compounds in a wide range of SARS-CoV-2 related assays using ML. In terms of public health, AI is also used to (1) predict the dynamics of infectious diseases and the effects of interventions, (2) detect and monitor outbreaks, and (3) detect infectious diseases in real time. From the above, it can be seen that AI is effectively used in various aspects of COVID-19 countermeasures. Due to space limitations, we were unable to describe them in this review, but notable contributions have also been made through the use of distributed artificial intelligence and agent-based models [138,139,140]. We note that, from the perspective of preventing infectious diseases, it is necessary to reduce human contact and unnecessary human flow, which is why information and communication technology in social life is advancing worldwide [141,142,143]. AI is expected to become increasingly important in the future, as the digitalization of data will increase along with the shift to information and communication technology in the medical field.

The spread of COVID-19 is having a major impact on the world, and the role of science and technology in overcoming infectious diseases is becoming increasingly important. Many countries have fallen into a lockdown situation due to the spread of COVID-19 infection. We believe that to free the people of the world from the threat of COVID-19, it will be necessary to continue to fight COVID-19 with the best of the world’s science and technology, and the AI approaches described in this review are expected to be utilized as fundamental techniques.

Although the potential of AI is very high, there are some problems that need to be considered and addressed appropriately. The first main problem is overfitting, which refers to the state where the system is trained to fit the training data but does not fit the test data, resulting in poor generalization performance [15]. COVID-19 is a new disease, and less than two years have passed since the first case was reported. Because the amount of data accumulated so far may not be sufficient in some cases, generalization performance needs to be assessed carefully. The second main problem is interpretability and explainability, that is, the development of explainable AI. From the perspective of building a relationship of trust between clinicians and AI, it is necessary to make efforts to present the reasons and rationale for decisions made by AI in an easy-to-understand manner. In a study by Oh et al., research results using Grad-CAM were presented [36]. The third main problem is related to domain shift, and it has been reported that the accuracy of predictions made using AI models is good in single-center studies, but in multi-center studies, high accuracy is not always obtained in all facilities [15,144,145]. This may be due to differences in the manufacturer, model number, and protocols of the medical devices used at each facility. We believe that it is essential to build a platform where the capabilities of the AI itself do not differ between facilities by utilizing techniques such as fine-tuning and domain adaptation [22,146,147,148]. Besides the main problems mentioned above, one must also be aware of the fact that COVID-19 is a new disease and therefore information is continuously being updated. For example, it should be noted that (a) information on viral mutations, (b) information on treatments, and (c) information on the composition of the affected community will continue to be updated, so it should be noted that AI trained on old information may not perform well.

Although some problems with the use of AI have been observed, as described above, it is undeniable that its potential is high. Therefore, we hope that AI will continue to make a significant contribution to the diagnosis and treatment of COVID-19 while innovations to compensate for its shortcomings are made.

## 6. Conclusions

There have been several epidemics in the past. Based on human history, we will not be able to completely eradicate harmful viruses owing to mutations and drug resistance. Needless to say, it is important to continually advance and keep up with the latest developments in viral infection. As we have reviewed in this article, there are several advantages to using AI to fight against COVID-19, including acceleration of the discovery of valuable vaccines or drugs to prevent pandemics and facilitation of the diagnosis of diseases and infections. Moreover, explainable AI should accelerate the implementation of AI in society, which can help improve the quality of decision-making in hospitals.

## Data Availability

Not applicable.
